# Split Dose of Prednisolone in the Treatment for Erythema Nodosum Leprosum: A Case Series

**DOI:** 10.7759/cureus.60888

**Published:** 2024-05-23

**Authors:** Swetalina Pradhan, Gaurav Dash, Debopriya Paul, Rashid Shahid

**Affiliations:** 1 Dermatology, All India Institute of Medical Sciences, Patna, Patna, IND

**Keywords:** pustular., necrotic, steroids, split dose of prednisolone, erythema nodosum leprosum, hansens, leprosy

## Abstract

Background

Erythema nodosum leprosum (ENL) is an immune complex-mediated reaction that clinically presents as tender erythematous evanescent nodules, mostly associated with systemic symptoms. Oral prednisolone is the drug of choice, with doses ranging from 0.5 to 1 mg/kg. Some cases may develop new lesions and systemic symptoms despite 1 mg/kg prednisolone, and in ideal practice, physicians escalate the prednisolone dose for immediate arrest of inflammation to prevent complications. However, a high dose of prednisolone has more side effects in the long term and causes more immunosuppression.

Methods

In cases of ENL, those not responding to a conventional once-daily regimen were given a split dose of oral prednisolone instead of increasing the dose. They were followed up for response, and serum cortisol was measured to see for hypothalamic-pituitary-adrenal (HPA) axis suppression.

Results

Eight cases of ENL (three nodular, three necrotic, one pustular, and one nodulcerative) had a dramatic response to split-dose therapy without any relapse and HPA axis suppression.

Conclusion

A split-dosing regimen can be a good treatment option in ENL with better control, less steroid dependency, and a lower relapse rate.

## Introduction

Leprosy is a chronic granulomatous disorder primarily affecting the skin and nerves, sometimes associated with acute exacerbation-like reactions (types 1 and 2) [1]. Erythema nodosum leprosum (ENL) is characterized by sudden crops of evanescent, erythematous, tender nodules associated with symptoms like fever and organ involvement like the kidney, testes, and eye in severe cases [2-4]. So, early arrest of the disease is essential to prevent complications. Prednisolone is considered the first line of treatment, with a dosage ranging from 0.5 to 1 mg/kg [5,6]. In the event of the appearance of new lesions, the dose has to be increased as per standard protocol until there is complete control of ENL. However, this increases the risk of long-term side effects. Here, we describe eight cases of ENL controlled with the split-dose regimen of prednisolone without any relapse.

## Materials and methods

Study design and setting

This observational study consisting of case series was conducted in the dermatology outpatient clinic of All India Institute of Medical Sciences, Patna, India from July 2023 to December 2023. The diagnosis of LL with ENL was mostly clinical, with confirmation by histopathology in a few cases.

Inclusion criteria

Cases of LL with ENL (naive or already on multidrug therapy) ranging in age from 18 to 60 were not responding to the conventional once-daily regimen of oral prednisolone.

Data collection

The demographic details like duration of disease, treatment received, spectrum of leprosy, type of ENL, number of ENL episodes, timing of eruption of ENL lesions, presence of neuritis, and systemic symptoms of all the cases were collected in the table. They were followed up for response, and serum cortisol was measured to see for hypothalamic-pituitary-adrenal (HPA) axis suppression. Data like the initial treatment response and timing of the split dose of prednisolone were also collected in the table.

## Results

**Figure 1:**
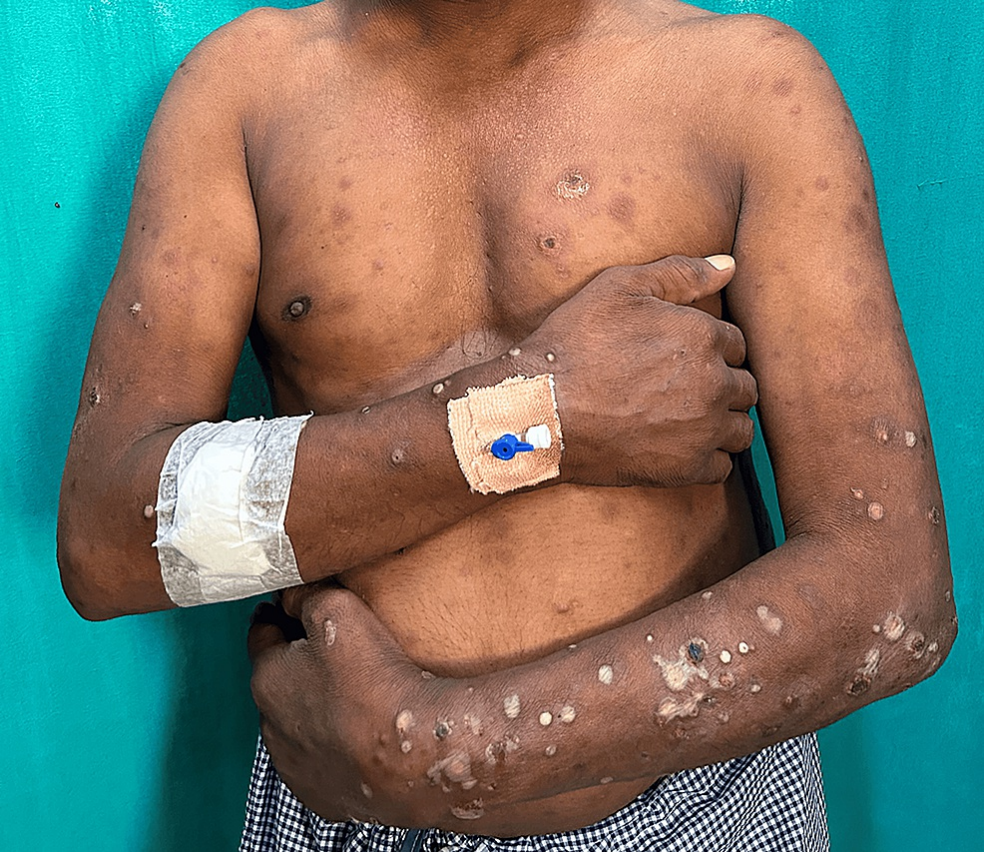
Patient with lepromatous leprosy with pustular presentation of ENL (Case 2) ENL, erythema nodosum leprosum

**Figure 2:**
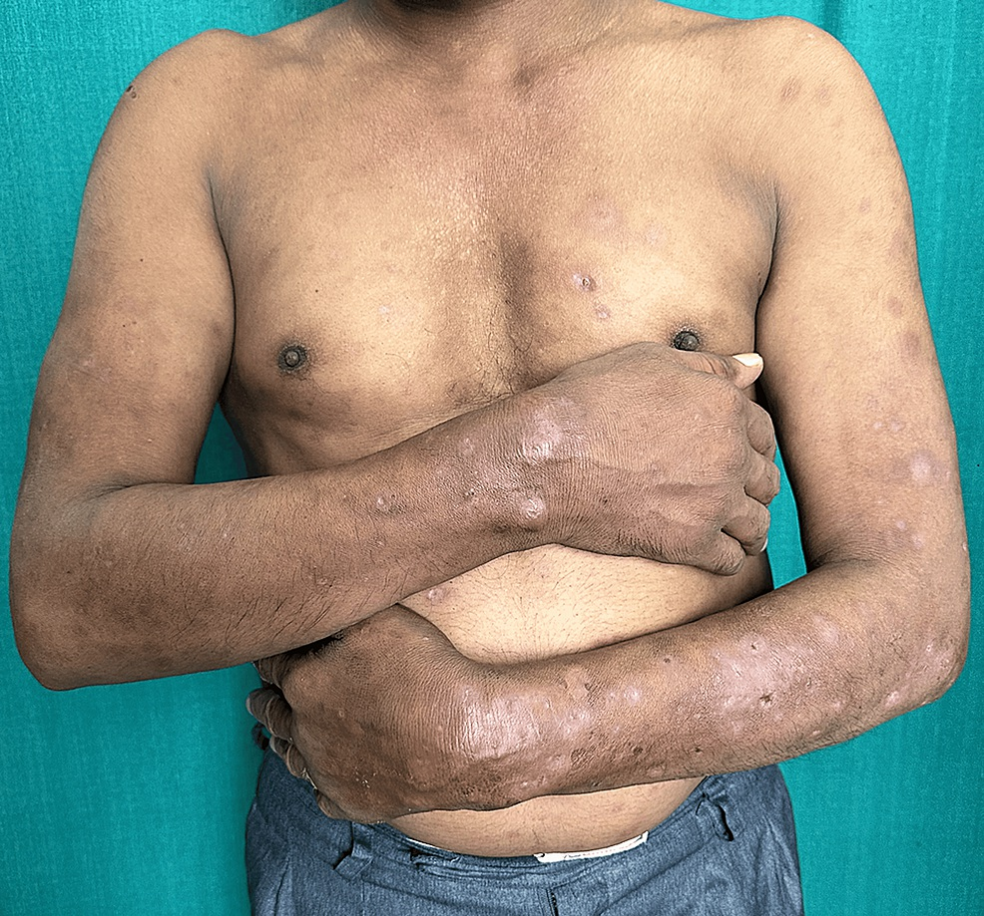
Complete subsidence after treatment in the pustular presentation of ENL (Case 2) ENL, erythema nodosum leprosum

Six male and two female patients with lepromatous leprosy ranging in age from 35 to 57 years presented with ENL in the form of sudden onset of skin lesions like nodules and pustules with systemic features like fever and joint pain (Cases 1-8), redness of the eyes (Cases 1-3, 6, and 7), testicular pain (Case 3), and painful lymphadenopathy (Case 5). The disease duration ranged from four to 24 months. Three patients (Cases 4, 6, and 7) presented with classical nodular lesions, while another three patients (Cases 1, 3, and 8) presented with necrotic ENL lesions. One patient (Case 2) presented with pustular ENL (Figure 1), and in another patient (Case 5), both nodular and ulcerative ENL lesions were present. The demographic details like duration of disease, treatment received, spectrum of leprosy, type of ENL, number of ENL episodes, presence of neuritis, and systemic symptoms of all the cases are described in Table 1. Routine hematological parameters revealed anemia in most of the cases (Cases 1-3, 5, and 8), hepatitis (Case 2), and proteinuria (Cases 1, 2, and 5). In all patients, we started oral prednisolone (1 mg/kg) according to their body weight, along with thalidomide 200 mg daily. Despite continuing oral prednisolone for an adequate period (seven days), all the patients continued to have new crops of ENL lesions, mostly in the evening and nighttime, so instead of increasing the dose, we split the dose of prednisolone into two divided doses, with which a significant response was obtained in all the cases within a short duration (Figure 2). Table 2 demonstrates the prior treatment given, the initial treatment response, the timing of the eruption of ENL lesions, the timing of the split dose of prednisolone, the response to split-dose therapy, and the serum cortisol level during follow-up in detail. All patients had normal serum cortisol during follow-up while on a split dose. Tapering of the steroid was done by 5 mg every two weeks in all patients, with the evening dose first followed by the morning dose. Follow-up serum cortisol was done in all patients when they reached only the morning dose (completion of evening dose tapering), and no patients were found to have HPA suppression.

**Table 1:** Demographic details ENL, erythema nodosum leprosum; LL, lepromatous leprosy; MB(A), multidrug therapy multibacillary adult; MDT, multidrug therapy

Case	Age and sex	Duration of disease	Leprosy spectrum	Treatment undergoing	ENL type	Number of ENL episodes	Neuritis	Systemic symptoms
1	35, female	10 months	LL	MDT MB(A) for 2 months	Necrotic	1	No	Fever, joint pain, and redness of the eyes
2	51, male	6 months	LL	No treatment	Pustular	1	No	Fever, joint pain, and redness of the eyes
3	37, male	9 months	LL	MDT MB(A) for 4 months	Necrotic	3	No	Fever, joint pain, redness of the eyes, and testicular pain
4	57, male	5 months	LL	MDT MB(A) for 3 months	Nodular	2	No	Fever and joint pain
5	45, female	11 months	LL	No treatment	Nodular and ulcerative	4	No	Fever, joint pain, and painful axillary and inguinal lymphadenopathy
6	39, male	7 months	LL	MDT MB(A) for 9 months	Nodular	3	Yes	Redness of the eyes, fever, and joint pain
7	29, male	20 months	LL	MDT MB(A) for 16 months f/b Alternate MDT for 6 months	Nodular	4	Yes	Fever, joint pain, and redness of the eyes
8	40, male	4 months	LL	No MDT	Necrotic	3	Yes	Fever and joint pain

**Table 2:** Treatment details

Case	Treatment given (1 mg/kg of prednisolone)	Treatment response	Timing of the onset of a new lesion and fever	Split-dose timing	Response to a split dose	Serum cortisol (5-25 mcg/dL) during follow-up
1	50 mg of prednisolone with thalidomide	The patient was still getting new lesions even after one week	1 AM	8 AM: 30 mg; 8 PM: 20 mg	After 48 hours, there were no new lesions	14 mcg/dL
2	50 mg of prednisolone with thalidomide	New pustules with high-grade fever even after five days of prednisolone	10 PM	8 AM: 30 mg; 8 PM: 20 mg	No new lesions after 24 hours; patient afebrile after two days	18 mcg/dL
3	60 mg of prednisolone with thalidomide for one month	Still getting ENL lesions intermittently	7 to 8 PM	7 AM: 30 mg; 7 PM: 30 mg	No new lesions after one week	12 mcg/dL
4	65 mg of prednisolone with thalidomide for three weeks	Still getting nodules with fever and joint pain	4 AM	8 AM: 35 mg; 8 PM: 30 mg	No new lesions after three days	15 mcg/dL
5	40 mg of prednisolone with thalidomide for four weeks	Still getting nodules with ulcers along with fever, joint pain, and redness of the eyes	10 to 11 PM	8 AM: 20 mg; 8 PM: 20 mg	No new lesions after a week	20 mcg/dL
6	50 mg of prednisolone with thalidomide for four weeks	New nodules at a two- to three-day interval	5 to 6 AM	8 AM: 30 mg; 8 PM: 20 mg	No new lesions after seven to 10 days	10 mcg/dL
7	60 mg of prednisolone with thalidomide for four months	New nodules after four to five days	5 to 6 PM	8 AM: 40 mg; 8 PM: 20 mg	No new lesions after 48 hours	19 mcg/dL
8	50 mg of prednisolone with thalidomide for one month	New nodules after one to two days	9 to 10 PM	8 AM: 30 mg; 8 PM: 20 mg	No new lesions after three days	12 mcg/dL

## Discussion

ENL, usually occurring in the lepromatous pole (borderline lepromatous and lepromatous leprosy), is characterized by crops of tender nodules. However, bullous, pustular, ulcerated, hemorrhagic, and erythema multiforme-like lesions are rarely described in the literature [7-9]. It may be classified as acute, recurrent, or chronic. Acute ENL is defined as a single episode lasting less than 24 weeks, whereas if it is more than 24 weeks requiring continuous treatment or any treatment-free period of 27 days or less, it is chronic ENL. Recurrent ENL is characterized by repeated episodes occurring after 28 days of stopping treatment [6]. Systemic involvement also occurs in the form of lymphadenitis, neuritis, iridocyclitis, arthritis, synovitis, myositis, epididymo-orchitis, and glomerulonephritis [7,8]. Thus, early control of ENL is required to prevent complications like organ damage and loss of nerve function [7]. Three patients (Cases 1, 3, and 8) presented with necrotic ENL lesions with systemic involvement like fever, joint pain, anemia, proteinuria, redness of the eye, and testicular pain. The remaining patients (Cases 4-7) presented with classical nodular ENL lesions with systemic involvement like fever, joint pain, anemia, proteinuria, redness of the eye, and painful lymphadenopathy. Another patient (Case 2) presented with pustular ENL lesions with systemic involvement like fever, joint pain, anemia, redness of the eye, proteinuria, and hepatitis.

Corticosteroids such as prednisolone, the first-line treatment, rapidly arrest the inflammation of ENL. In usual practice, the dose has to be hiked if ENL does not get controlled [3]. However, high-dose prednisolone has side effects like hyperglycemia, hypertension, steroid dependency, cataracts, osteoporosis, and complications secondary to immunosuppression [10,11]. Serum cortisol levels reach their peak in the morning (8 AM) and gradually decline to the minimum at around 4 AM [12]. The rationale behind once-daily morning prednisolone is to cause minimal HPA suppression [13]. However, in situations where the demand for immunosuppression is greater, systemic steroids can be given in twice-daily doses. There are studies where split doses were given in relapsing nephrotic syndrome with remission without any side effects [14]. ENL, being an immune-complex-mediated lepra reaction, endangers almost all systemic organs of the body. Hence, in such cases, our goal of treatment should be immediate control of ENL with judicious use of steroids causing minimal side effects. Because of low cortisol in the evening, ENL lesions aggravate in the evening. This is the reason why a divided dose is recommended in ENL. As it is a vasculitis disorder, a divided dose is also recommended, and an evening dose is recommended. Initially, we started oral prednisolone at 1 mg/kg once daily in the morning for all our patients as per guidelines. But still, new crops of lesions kept occurring even after continuing oral prednisolone for a few weeks in our cases. It was mostly during the evening and night, which is expected due to low serum cortisol levels during this period. So, instead of escalating the dose further, we started a split-dosing regimen where the initial single dose was divided into morning and evening dosages at an interval of 12 hours. The timing differed from patient to patient, depending on the timing of the eruption of new lesions. ENL was well controlled in all these patients within a short period of time. The dose was tapered gradually (the evening dosage was tapered first, followed by the morning) after the ENL lesions and systemic symptoms had subsided completely. Tapering was done by 10 mg every 15 days up to 20 mg, then by 5 mg every 15 days. There was no relapse while tapering. Additionally, none of the patients showed any signs or symptoms of HPA axis suppression, and all had normal serum cortisol levels during the follow-up period after the completion of evening dose tapering. Splitting the dose of steroids is a wiser idea for better control of ENL, as it has a tendency to occur in the evening. Hence, it is a good option in cases not responding to the conventional once-daily 1 mg/kg morning dose of prednisolone, which not only decreases long-term side effects but also decreases dependency on steroids. On review of the literature, we could find only one case report with a split-dose regimen in ENL documented to date [15].

## Conclusions

A split-dosing regimen is a very novel and effective option in ENL, especially for those not responding to the conventional once-daily regimen. It has better control with dramatic improvement without relapse. Also, there is less steroid dependency, and no HPA axis suppression was observed.
